# Long-Term Exposure to Real-Life Polyethylene Terephthalate Nanoplastics Induces Carcinogenesis In Vitro

**DOI:** 10.1021/acs.est.5c01628

**Published:** 2025-06-02

**Authors:** Javier Gutiérrez-García, Raquel Egea, Irene Barguilla, Penny Nymark, Alba García-Rodríguez, Boris Guyot, Veronique Maguer-Satta, Ricard Marcos, Laura Rubio, Alba Hernández

**Affiliations:** † Group of Mutagenesis, Department of Genetics and Microbiology, Faculty of Biosciences, Universitat Autònoma de Barcelona, Cerdanyola del Vallès, Barcelona 08193, Spain; ‡ CNRS UMR5286, 131822 Centre de Recherche en Cancérologie de Lyon, Lyon 69008, France; § Inserm U1052, Centre de Recherche en Cancérologie de Lyon, Lyon 69008, France; ∥ Institute of Environmental Medicine, 27106 Karolinska Institutet, Stockholm 17177, Sweden

**Keywords:** nanoplastics (NPLs), polyethylene terephthalate (PET), carcinogenicity, respiratory toxicity, chronic exposure, new approach methodologies (NAMs), human health risk

## Abstract

Micro/nanoplastics (MNPLs) are environmental contaminants originating mainly from plastic waste degradation that pose potential health risks. Inhalation is a major exposure route, as evidenced by their detection in human lungs, with polyethylene terephthalate (PET) among the most abundant particles in respiratory airways. However, the harmful effects of particle bioaccumulation remain unclear, as chronic effects are understudied. To assess long-term effects, specifically carcinogenic effects, BEAS-2B cells were exposed to PET-NPLs for 30 weeks. Genotoxicity, carcinogenic phenotypic hallmarks, and a panel of genes and pathways associated with cell transformation and lung cancer were examined and compared across three exposure durations. No significant effects were observed after 24 h or 15 weeks of exposure. However, a 30-week exposure led to increased genotoxic damage, anchorage-independent growth, and invasive potential. Transcriptomic analysis showed the upregulation of several oncogenes and lung cancer-associated genes at the end of the exposure. Further analysis revealed an increase in differentially expressed genes over time and a temporal gradient of lung cancer-related genes. Altogether, the data suggest PET-NPLs’ potential carcinogenicity after extended exposure, highlighting serious long-term health risks of MNPLs. Assessing their carcinogenic risks under chronic scenarios of exposure is crucial to addressing knowledge gaps and eventually developing preventive policies.

## Graphical abstract

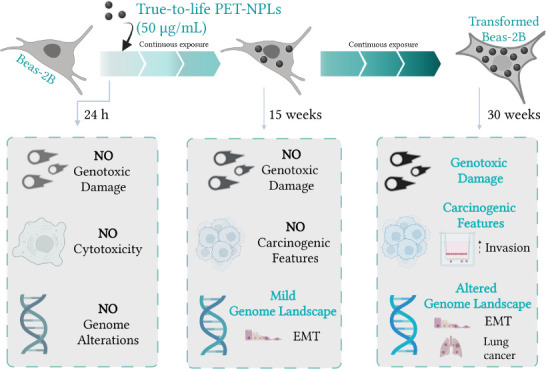

## Introduction

Plastics represent one of the most extensively utilized materials globally. As a result, global plastic production has escalated exponentially, now surpassing 400 million tons annually.^1^ Despite this extensive production, only approximately 9% of plastic waste is recycled, with the majority either being incinerated (12%) or persisting in the environment (79%).^2^ In the latter two scenarios, degradation of plastic polymers, whether through incomplete combustion or natural physicochemical processes, results in the generation and release of micro- and nanoplastics (MNPLs) into the environment.^3^ These particles can become airborne, facilitating their atmospheric transport over long distances, thereby rendering them ubiquitous in various ecosystems and posing a potential risk for human inhalation.^4,5^

The composition of airborne MNPLs is highly complex and heterogeneous, encompassing a broad spectrum of sizes, morphologies, polymer types, and surface charges, which presents significant challenges for accurate characterization.^6^ Moreover, technical constraints in the detection of nanoscale particles impede the identification and detailed analysis of smaller airborne polymers, specifically NPLs (<1 μm). Consequently, much of the current literature on airborne MNPLs predominantly addresses MPLs (1 μm < MPLs < 5 mm).^7^ Despite this limitation, available evidence indicates that the most detected polymers in atmospheric samples include polyethylene terephthalate (PET), polyethylene (PE), polypropylene (PP), and polystyrene (PS), with concentrations consistently higher in indoor environments compared to outdoor settings.^8,9^

Despite the limitations in the analysis of airborne MNPLs, their penetration into the human respiratory tract via inhalation has been directly demonstrated in multiple studies.^10^ Analysis of sputum samples, lung tissue digests, and bronchial and nasal lavage fluids has confirmed the presence of various MPLs in the human respiratory system.^4,11,12^ Among the MPLs detected in human lung tissues, PET particles are some of the most frequently observed.^9^ PET is predominantly used in the production of beverage bottles, including those for water, soft drinks, and juices, representing the largest share of global PET production.^1^ Another significant application of PET is in the textile industry, where it is used in the form of polyester fibers, which, along with other synthetic fibers, are considered the primary source of airborne MNPLs.^9^ Additionally, PET is a key component in the manufacturing of disposable face masks and tobacco filters, further increasing the potential for inhalation exposure.^7,13^

Among the potential adverse effects of PET-MNPLs on the respiratory system, their capacity to promote cellular transformation has raised significant concerns.^14^ The efficient cellular uptake of these particles, attributed to their small size, in conjunction with their biopersistence and the intracellular effects documented to date—including genotoxicity—may contribute to carcinogenesis.^15,16^ Noteworthily, different human studies have examined the potential association between PET microplastics, including synthetic fiber mixtures of varying sizes, and an increased risk of lung cancer in human populations.^17,18^ The work of Mastrangelo et al.^17^ concluded that high-frequency exposure of textile industry workers to PET correlates with an increased incidence of lung cancer. Similarly, Chen et al.^18^ found a higher detection rate of microfibers, including PET-MPLs, in lung tissues of patients with nonsmall cell lung cancer compared to unaffected individuals.

Although these human studies have provided important insights into the association between MPL exposure and lung cancer, no toxicological studies have been conducted to investigate the specific causal linkages between PET exposure and lung cancer. Furthermore, no human studies have yet assessed the carcinogenic potential of nanosized PET, despite evidence suggesting that the unique characteristics of nanoparticles—such as their ability to penetrate cellular membranes and their increased reactivity—indicate a greater toxicological potential compared to larger counterparts. In this regard, Domenech et al.^14^ recently utilized PET-NPLs derived from the degradation of water bottles to evaluate their cellular transforming potential through an in vitro colony transforming assay (CTA) employing Bhas-42 cells. Notably, this study identified PET-NPLs as potential promoters of the carcinogenic process. Although the Bhas-42 CTA is an OECD-standardized method that provides valuable data for carcinogenicity screening, it is a single-endpoint analysis that does not adequately capture the complex, multistep nature of carcinogenesis and fails to provide insights into the underlying molecular mechanisms of action. To overcome these limitations, there have been great advances in alternative testing methodologies and frameworks to support their use for regulatory purposes. Hypothesis-driven integrated testing strategies (ITSs) have been proposed as a solution to provide a structure for the integration of data derived from multiple sources (different test methods, kinetics, exposure, computational toxicology, etc.) into one decision-making process. In addition, adverse outcome pathways (AOPs) provide a biological context and mechanistic rationale to support ITS.^19^ AOPs inform causal linkages between a molecular initiating event and a final adverse outcome. Although these frameworks have not yet been applied to MNPLs, their potential to enable efficient and timely hazard assessment has already been recognized.^20^

In this context, we aimed to investigate the carcinogenic potential of PET-NPLs, replicating the real-world scenario in which populations are exposed to subtoxic doses over extended periods. We developed an in vitro model using human lung epithelial BEAS-2B cells exposed to subtoxic doses for up to 30 weeks. Several phenotypic and molecular hallmarks of carcinogenesis were assessed and compared across multiple time points, offering an innovative approach for tracking the cell transformation process. Consequently, we provide insights into the carcinogenic potential of PET-NPLs using a system that aligns with the principles of ITS, with the potential to further the development of alternative approaches for MNPL risk assessment.

## Materials and Methods

### Obtention of PET-NPLs and Fluorescent iDye-PET-NPLs

PET-NPLs were obtained from bottle degradation following the protocol described by Villacorta et al.^21^ Briefly, PET powder was obtained by sanding pieces of water bottles with a diamond rotary burr, followed by sieving. The resulting powder was dispersed in preheated trifluoroacetic acid through stirring. Subsequent centrifugation was performed to separate the supernatant from the pellet, which was then resuspended in sodium dodecyl sulfate (SDS) and subjected to sonication. The emulsion was allowed to settle, after which the supernatant was washed with Milli-Q water and pure ethanol to remove SDS and then dried. The resulting PET-NPL powder was resuspended in Milli-Q water, sonicated, aliquoted into cryotubes, and immediately frozen in liquid nitrogen. Stock suspensions at a final concentration of 5 mg/mL were prepared following an adapted version of the NanoGenotox protocol.^22^ Briefly, a 6 mL dilution containing PET-NPLs, Milli-Q water, 0.05% sterile bovine serum albumin (BSA, CAS948468, Merck, Saint Louis, USA), and 30 μL of pure ethanol was sonicated for 16 min at 10° amplitude and fast frozen in liquid nitrogen. For the assessment of particle internalization, these particles were dyed following the protocol described by Villacorta et al.^23^ to obtain fluorescent iDye-PET-NPLs. Briefly, 10 mg of iDye-Pink (Rupert, Gibbon & Spider, Inc., Healdsburg, CA, USA) were added to a 5 mg/mL PET-NPLs suspension within a glass tube, achieving a final volume of 1 mL at the specified concentration. The mixture was subjected to a heating process at 70 °C for 2 h, followed by centrifugation in Amicon Ultra-15 centrifugal tubes (Merck, UFC9010D, Cork, Ireland) at 3220 *g* for 10 min. The flowthrough was discarded, and the residual particles were resuspended in Milli-Q water and centrifuged again. These resuspension and centrifugation processes were repeated seven times. The final particle concentration was resuspended in 1 mL of Milli-Q water. The use of this material has already been published in several studies and is expected to feature in upcoming publications, as it has been synthesized as a reference material for the EU project PLASTICHEAL (ref: 965196) for standardized use across the Consortium.

### Particle Characterization

The hydrodynamic size and Z-potential of the PET-NPLs were measured in triplicate using a Malvern Zetasizer Nano ZS ZEN3600 (Malvern Instruments, Malvern, UK), employing dynamic light scattering (DLS) for size analysis and electrophoretic light scattering (ELS) for surface charge determination. PET-NPL dispersions were prepared at a final concentration of 100 μg/mL, either in distilled water or Dulbecco’s modified Eagle’s medium (DMEM, Biowest, Nuaillé, France), for these measurements. The dry-state particle size was assessed via scanning electron microscopy (SEM; see Figure S1). For analysis, a single 10 μL drop of the PET-NPL suspension, at a concentration of 100 μg/mL, was applied to separate coverslips and allowed to dry overnight in a Petri dish covered with a lid to prevent contamination. The nanoparticle lipid (NPL) samples were subsequently examined using SEM (Zeiss Merlin, Zeiss, Oberkochen, Germany). The obtained SEM images were analyzed to determine the particle size distribution, specifically by measuring the Martin diameter using ImageJ software version 1.8.0_172. The mean particle diameter was determined by analyzing 100 randomly selected nanoparticles using ImageJ software with the Fiji extension (v. 2.16.0).

### Cell Culture Conditions and Long-Term PET-NPLs Exposure

The BEAS-2B human bronchial epithelial cell line (ATCC, CRL-9609) was cultured in DMEM supplemented with 10% fetal bovine serum (FBS) (Biowest, Nuaillé, France), 1% nonessential amino acids (Biowest), and 2.5 μg/mL Plasmocin (InvivoGen, San Diego, CA, USA). Cells were maintained in *T*-75 flasks (LabClinics, Barcelona, Spain) in a humidified incubator at 37 °C with 5% CO_2_ and 95% air. Subculturing was performed when cells reached approximately 80% confluence using trypsin (Biowest) for detachment. The carcinogenic effects of environmental pollutants arise from sustained exposure over long periods, making chronic exposure particularly relevant in in vitro studies. Therefore, a novel exposure approach consisting of sustained exposures throughout extended periods of time was followed in this study. Briefly, BEAS-2B cells were chronically exposed for 30 weeks to 50 μg/mL of PET-NPLs in *T*-75 flasks by passaging the cells weekly at a seeding density of 4000 cells/cm^2^, and by replacing the cell culture medium every 3–4 days with fresh medium containing the selected concentration of PET-NPLs. Importantly, nonexposed control cells were maintained in parallel for the same number of weeks to discriminate the effects induced by continuous passaging. A cell biobank representative of the whole exposure period was established to facilitate accessibility to the cellular samples for subsequent studies.

### Particle Internalization

To determine the ability of BEAS-2B cells to internalize PET-NPLs, confocal microscopy and flow cytometry techniques were used. Visual detection and localization of PET-NPLs intracellularly were achieved by using confocal microscopy (Leica TCS SP, Wetzlar, Germany). For this, BEAS-2B cells (12 000 cells/well) were seeded in glass bottom microwell dishes (Ibidi, Gräfelfing, Germany) and treated with 50 μg/mL iDye-labeled PET-NPLs for 24 h. Following treatment, the medium was refreshed, and the cells were stained for 10 min with Hoechst 33342 (Thermo Fisher Scientific, Waltham, MA, USA) to detect nuclei and with CellMask Deep Red plasma membrane stain (Life Technologies, Paisley, UK) to visualize cellular membranes, both at a 1:500 dilution in DMEM. Images were acquired using a Leica TCS SP5 confocal microscope and processed with IMARIS software. Additionally, flow cytometry was employed to quantify the internalization of iDye-labeled PET-NPLs. The cells (2 × 10^5^/well) were seeded in 24-well plates (LabClinics, Barcelona, Spain). Following exposure (50 μg/mL for 24 h), the cells were washed with 1× PBS, trypsinized, centrifuged, and analyzed using a CytoFlex cytometer (Beckman Coulter Inc., Carlsbad, CA, USA). Approximately 10 000 single-cell events were recorded per sample, and the percentage of cells containing iDye-PET-NPLs was determined.

### Cytotoxicity

The cytotoxic potential of PET-NPLs exposure in BEAS-2B cells over 24 h was assessed using a Beckman Coulter Z Series particle counter (Beckman Coulter Inc., Brea, CA, USA). In brief, 40 000 cells per well were seeded in 12-well plates, and after 24 h, the cells were exposed to increasing concentrations of PET-NPLs (25, 50, 100, 150, and 200 μg/mL). Following 24 h of exposure, cell counts were obtained, and the percentage of viable cells was calculated by comparing the treated groups to nonexposed controls. Based on these results, a subtoxic dose of 50 μg/mL was selected for subsequent long-term exposure studies.

### Carcinogenesis Evaluation: Hallmarks of Carcinogenesis

A battery of carcinogenic features was selected as informative for the acquisition of the oncogenic phenotype induced by long-term PET-NPLs exposure. Details of the methodology and the rationale behind the selection can be found in the report by Barguilla et al.^24^

#### Genotoxic Damage

To assess DNA damage in BEAS-2B cells following exposure to PET-NPLs, the alkaline comet assay was performed following the recently published protocols.^25^ In brief, the cells exposed to 50 μg/mL PET-NPLs for 24 h, 15 weeks, and 30 weeks were seeded in 6-well plates (2 × 10^5^ cells/well), washed with 1× PBS, detached using trypsin (37 °C, 5 min), and pelleted by centrifugation (200 *g*, 8 min). The pellets were resuspended in 1× PBS to a final concentration of 1 × 10^6^ cells/mL. Cell suspensions were mixed at a 1:10 ratio with 0.75% low melting point agarose (37 °C), and 10 μL droplets were placed in triplicate on GelBond films (Life Sciences, Vilnius, Lithuania). The films were incubated overnight in lysis buffer at 4 °C, washed for 5 min in electrophoresis (EF) buffer, and then incubated with fresh EF buffer for 35 min at 4 °C to allow DNA unwinding. EF was performed at 20 V and 300 mA for 20 min at 4 °C. Following EF, several PBS washes and distilled water rinses were conducted before cell fixation with absolute ethanol for 1 h. Finally, nuclei were stained with SYBR Gold (1:10 000 in Trizma base 10 mM (Sigma-Aldrich, Burlington, MA, USA) and EDTA 1 mM (VWR International, Radnor, PA, USA)) for 20 min at room temperature. Comet images were captured using an Olympus BX50 epifluorescence microscope (Olympus, Tokyo, Japan) at 20× magnification. DNA damage was quantified as the percentage of DNA in the tail using Komet 5.5 image analysis software (Kinetic Imaging Ltd., Liverpool, UK), with 100 cells analyzed per replicate. Cells treated with 200 μM methylmethanesulfonate (MMS, Sigma-Aldrich, St. Louis, MO, USA) for 30 min served as positive controls, while time-matched nonexposed cells were used as negative controls.

### Anchorage-Independent Cell Growth

The anchorage-independent growth ability of BEAS-2B cells chronically exposed to PET-NPLs was assessed by a soft agar assay. After 15 and 30 weeks of exposure, single-cell suspensions were prepared by passing the cells through a 40 μm mesh. A suspension of 1.25 × 10^5^ cells in 1.75 mL of DMEM containing 10% FBS and 2.5 μg/mL of Plasmocin was then mixed in a 1:1:1 ratio with 2× DMEM (containing 20% FBS, 2% nonessential amino acids, 2% l-glutamine (200 mM), and 2% penicillin–streptomycin) and with 1.2% bacto-agar (BD, Franklin Lakes, NJ, USA). Triplicate samples of 4 × 10^4^ cells each were plated into 6-well plates, with 4.5 mL of the cell mixture dispensed into each well on top of a 0.6% base agar layer supplemented with 2× DMEM. Once the agar solidified, the plates were incubated for 21 days. Colonies were stained using 1 mg/mL (2-*p*-iodophenyl)-3-(*p*-nitrophenyl)-5-phenyl tetrazolium chloride (INT; Sigma-Aldrich, Burlington, MA, USA). Plates were scanned using an HP Scanjet G4050 for colony quantification, and the number of colonies was determined using the OpenCFU colony counter software (version 3.9.0). Colonies with a radium size bigger than 26 μm were filtered, and values were annotated.

### Invasion Potential

Long-term exposed and time-matched control cells in *T*-25 flasks at 70% confluency were deprived of FBS for 24 h. Subsequently, 24 mm transwell inserts with 8 μm pore size polycarbonate membranes (Costar-Corning, Corning, NY, USA) were coated with 180 μL of a 1:2 dilution of Matrigel (Costar-Corning, Corning, NY, USA) in FBS-free DMEM containing 0.1% BSA. The Matrigel coating was allowed to dry in the cell incubator at 37 °C for 1 h. Following this, 2.5 mL of DMEM supplemented with 15% FBS was added to the bottom chamber of the transwell as the chemoattractant medium. A single-cell suspension of 4 × 10^5^ FBS-deprived BEAS-2B cells in 1.5 mL of FBS-free DMEM with 0.1% BSA was added to the top of the Matrigel-coated transwell membrane. The plates were then incubated at 37 °C for 48 h. To quantify cell invasion, the Matrigel layer and the noninvading cells on the top side of the transwell membrane were gently removed using a cotton swab. The cells that had migrated to the bottom side of the membrane were fixed with 1 mL of methanol (VWR International, Radnor, PA, USA) and stained with 0.2% crystal violet (Sigma-Aldrich, Burlington, MA, USA). After several washes, the membranes were dried and imaged using a Zeiss Observer A1 microscope (Boston Industries, Walpole, MA, USA). The proportion of invading cells was analyzed using ImageJ software.

### Transcriptomics

PET-NPLs-induced changes in the transcriptome of long-term exposed cells were determined to (i) explore whether the altered gene landscape supports the cells’ oncogenic transformation, (ii) establish potential carcinogenesis-associated modes of action, and (iii) identify new potential biomarkers directly connected to PET-NPLs-induced carcinogenesis.

### Total RNA Extraction and RNA-Seq

RNA from cells exposed to PET-NPLs for 24 h, 15 weeks, and 30 weeks, as well as from the corresponding passage-matched control cells, was extracted using TRI Reagent (Sigma-Aldrich, Burlington, MA, USA) according to the manufacturer’s instructions. DNA contamination was removed using RNase-free DNase I (Turbo DNA-free Kit, Life Technologies, Carlsbad, CA, USA). RNA samples were quantified and sent to Macrogen (Seoul, Korea) for RNA sequencing with the Illumina platform, with a read length of 2 × 150. A mean of 40 million reads per file was obtained and analyzed.

### Data Analysis

Transcriptomic analyses for RNA-Seq data were performed using R (v. 4.3.2) and RStudio (v. 2023.06.1) software.^26,27^ Raw FASTQ files were quality-filtered by the Rfastp package.^28^ Illumina sequence adapters were removed, and reads were trimmed and discarded if they were below 20 bases in length. Filtered reads were mapped to the human genome GRCh38, retrieved from GENCODE, and counted using the Rsubread package.^29^ Lowly expressed genes from the resulting count R objects were filtered by the filterByExpr function, and the remaining gene counts were normalized by the calcNormFactors function, both from the edgeR package^30^ with default parameters. Surrogate variant analysis was carried out with sva package^31^ and differentially expressed genes (DEGs) were obtained through the limma^32^ and voom^33^ packages by contrasting the data from the 24 h, 15 weeks, and 30 weeks PET-NPLs exposed samples versus their respective nonexposed controls. To determine the enriched functional terms, over-representation analysis (ORA) and gene set enrichment analysis (GSEA) were performed by the clusterProfiler package^34^ using different collections as models: Gene Ontology (GO), Disease Ontology (DO), Kyoto Encyclopedia of Genes and Genomes (KEGG), and the MSigDB hallmark collection (h). All analyses were performed with default parameters for all databases except GO, where the set size argument was configured to 800. Data visualization was performed by the ggplot2 package.^35^

### Gene Panels

Gene panels of carcinogenic-related pathways were collected from different databases, including Hallmarks MSigDb Collections and HistoneDB 2.0.^36^ An additional gene panel was created by using the Enrichr webpage to identify widely repeated genes in lung carcinoma, along with other genes compiled from the bibliography.^37^ All gene panels were intersected with genes exhibiting consistent or increased expression trends between 15 and 30 weeks of exposure.

## Results and Discussion

### PET-NPLs Characterization

Despite the prominent importance of PET as an airborne contaminant, most toxicity studies to date have been performed using commercially available materials of other polymer types, mainly pristine monodisperse polystyrene (PS) with uniform shapes and sizes, which are not representative of environmental samples. In contrast, in this study, we employed real-life PET-NPLs obtained from the degradation of plastic water bottles, exhibiting physicochemical properties that more closely resemble those of naturally occurring MNPLs. As shown below, these particles are polydisperse, displaying a diverse range of sizes and morphologies, and more accurately reflecting the complexity of environmental MNPL exposure.

The size distribution, Z-potential, and shape of the obtained PET-NPLs were characterized using a Zetasizer to measure the hydrodynamic size and Z-potential, while scanning electron microscopy (SEM) was used to determine their shape and dry-state dimensions, in agreement with the previously reported characterization of the particles obtained following the same procedure.^21^ SEM revealed that PET-NPLs exhibit an irregular shape with an average diameter of approximately 176 nm and a broad distribution of 10–1000 nm (PDI of 0.67). As expected, size variations were observed depending on the methodological approach used, with DLS giving average size values of 420 nm for PET-NPLs suspended in cell culture media (with a PDI value of 0.73 and a zeta potential of −15.2 mV). All obtained data are summarized and available in the Supporting Information (Figure S1).

### PET-NPLs are Not Cytotoxic in BEAS-2B Cells

To evaluate the potential cytotoxic effects of PET-NPLs on BEAS-2B cells, cell viability was assessed after 24 h of exposure to a range of doses (10, 25, 50, 100, and 200 μg/mL). As shown in Figure 1A, no significant cytotoxicity was observed at any of the tested concentrations. Consistent findings across several studies have demonstrated a lack of acute cytotoxicity of PET-NPLs from the same origin when applied to different cell lines and in vivo models. Thus, negative results were obtained when treating alveolar MH-S cells, human nasal epithelial cells (HNEpCs), THP-1 monocytes, and TK6 lymphoblasts.^38,39^ Likewise, in vivo experiments involving Drosophila melanogaster exposed to the same PET-NPLs throughout the larval stage reported no significant reduction in survival rates.^40^

**Figure 1:**
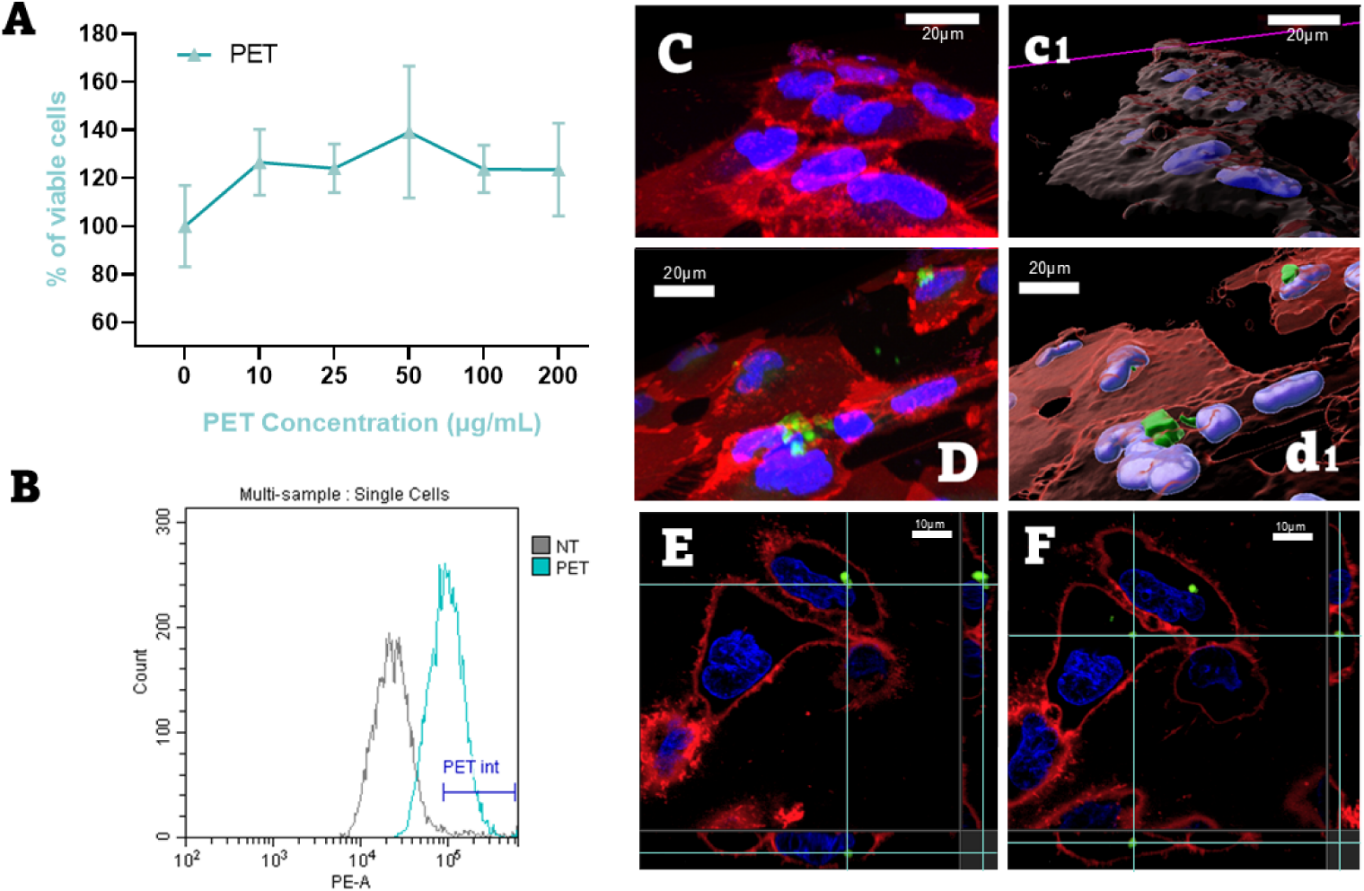
Interaction of PET-NPLs with BEAS-2B cells. (A) Cytotoxicity assessment of PET-NPLs in BEAS-2B cells. (B) Flow cytometry analysis showing fluorescence intensity (PE-A) in the cells as a measure of cellular interaction. The gray curve represents the negative control (NT), while the blue curve corresponds to PET-NPL-treated cells. (C–F) Confocal microscopy images: (C) BEAS-2B cells without treatment and (c1) the corresponding IMARIS 3D reconstruction; (D) BEAS-2B cells exposed to PET-NPLs for 24 h and (d1) the corresponding IMARIS 3D reconstruction. (E, F) Orthogonal views illustrating localization of PET-NPLs inside the cells.

If we focus on the cellular model instead of the material—as we acknowledge that each cellular type may exhibit a differential cellular response—other studies using pulmonary cells have reported mixed results regarding the cytotoxicity of PET-NPLs from different sources. For instance, Ji et al.^41^ observed cytotoxicity in TC-1 mouse lung epithelial cells following a 24 h exposure to PET-NPLs at a concentration of 100 μg/mL. Similarly, Zhang et al.^42^ reported a dose-dependent decrease in the viability of A549 cells exposed to PET-NPLs for 24 h, with reduced viability at concentrations higher than 100 μg/mL. In contrast, Alzaben et al.^43^ found that synthetically produced PET-NPLs did not induce cytotoxicity in A549 cells after 24 h of exposure at concentrations up to 125 μg/mL, which is consistent with our own findings.

Considering the absence of acute cytotoxicity at concentrations below 100 μg/mL observed both in this study and in others using comparable PET-NPL materials and cell targets, a noncytotoxic concentration of 50 μg/mL was selected for subsequent long-term exposure experiments. Additionally, kinetic data, as detailed below, were considered part of the selection criteria.

### Kinetics of PET-NPLs Uptake and Distribution in BEAS-2B Cells

To better understand the interaction between PET-NPLs and BEAS-2B cells, two complementary techniques—flow cytometry and confocal imaging—were employed, utilizing fluorescently labeled iDye-PET-NPLs. Both approaches indicated a moderate level of particle internalization, with flow cytometry showing that 60% of the cells contained particles after 24 h of exposure (Figure 1B). Interestingly, the kinetics analysis enabled a qualitative assessment of the effective concentration, which appeared lower than expected. This highlights the importance of considering effective concentrations in the MNPL field, as discrepancies between the administered concentration and the actual concentration at the cellular level are likely to occur, potentially resulting in lower-than-anticipated cellular exposure. The observed kinetics support the suitability of the selected concentration for chronic exposure studies (50 μg/mL), as it represents the minimal applied concentration that ensures sufficient particle uptake without inducing observable toxicity. Since not all cells are effectively exposed, this concentration also allows for potential bioaccumulation under prolonged exposure conditions. Additionally, confocal microscopy provided insights into the spatial distribution of the particles, revealing their proximity to the nuclear membrane (Figure 1C,D).

A high capacity for cellular uptake has been observed across various cellular models exposed to PET-NPLs from the same source, albeit at higher concentrations (typically 100 μg/mL). Tavakolpournegari et al.^39^ demonstrated through flow cytometry that 100% of alveolar macrophage MH-S cells internalized particles after a 24 h exposure, highlighting their phagocytic capability. Similarly, monocyte-derived THP-1 cells showed high PET-NPL internalization after only 3 h of exposure, as confirmed by TEM, confocal microscopy, and flow cytometry. In human primary nasal epithelial cells, internalization of PET-NPLs was also observed, with confocal microscopy suggesting their localization within endosomes near or inside the nucleus.^38^ Furthermore, confocal microscopy revealed the presence of PET-NPLs within the cytoplasm and close to the nucleus of Bhas-42 cells after both 4 and 10 days of treatment.^14^ Beyond cell cultures, Arribas et al.^44^ reported PET-NPL internalization in human blood cells after 24 h of whole blood exposure, as assessed by flow cytometry. Finally, in vivo uptake was evaluated in D. melanogaster following ingestion of PET-NPLs, with TEM and confocal microscopy analyses revealing efficient particle internalization by enterocytes and subsequent translocation to hemocytes.^40^ Supporting these findings, additional studies utilizing PET-NPLs of alternative origins have demonstrated high levels of cellular uptake across different cellular models. Specifically, epithelial alveolar A549 cells,^42^ intestinal Caco-2 cells,^45,46^ alveolar macrophage RAW 264.7 cells,^47^ and the hepatocarcinoma HepG2 cell line^48^ have all shown a remarkable capacity to internalize PET particles. Collectively, the findings indicate the efficient cellular uptake of PET-NPLs, presumably facilitated by their small size, favorable physicochemical properties, and hydrodynamic behavior in the different biological matrices tested.

### Carcinogenic Potential of PET-NPLs Long-Term Exposure

At present, in vivo rodent bioassays remain the gold standard for carcinogenicity assessment, with two-year studies, as outlined by OECD Test Guidelines 451 and 453, being the conventional method.^49,50^ These assays involve prolonged exposure and rigorous monitoring for toxicity and tumor development but are hindered by their time-consuming nature, high costs, and extensive use of animals, posing challenges for large-scale testing. This highlights the urgent need for alternative in vitro models to evaluate carcinogenic risks efficiently and ethically.^51,52^

To address this critical gap, Domenech et al.^14,49^ conducted a carcinogenicity assessment of NPLs using the standardized in vitro CTA with Bhas-42 cells, following OECD guidelines (GD 231). This assay differentiates between tumor initiators and promoters, evaluating genotoxic and nongenotoxic mechanisms, respectively.^53,54^ PET-NPLs notably induced a significant increase in transformed foci during the promotion phase of the assay at higher concentrations (200 μg/mL), indicating their potential role as tumor promoters.^14^

Building on this concerning finding, we expanded our investigation of PET-NPLs’ carcinogenic potential using a novel in vitro system designed to mimic chronic exposure of relevant target cells to subtoxic doses over extended periods.^55–58^ This system enables evaluation of key carcinogenic biomarkers, including genotoxicity, anchorage-independent growth, and invasive potential, throughout the entire exposure period.^24^ Thus, we continuously exposed BEAS-2B cells to 50 μg/mL PET-NPLs for up to 30 weeks and evaluated key carcinogenic effects, including some previously outlined in an AOP on lung cancer.^59^ Importantly, this system facilitates investigation into the molecular mechanisms underlying the observed effects and additionally provides a basis for the development of an integrated testing strategy (ITS) for NPLs, in line with what was described by Tollefsen et al. (2014).^19^

### Long-Term Exposure to PET-NPLs Leads to Genotoxic Effects

Genotoxicity is widely recognized as an early surrogate biomarker for carcinogenicity, to the extent that the OECD guidelines for chemical testing strongly recommend evaluating genotoxic potential due to its well-established link to cancer development.^15,49,60^ In this study, the genotoxic damage induced by PET-NPLs was assessed by using the comet assay after 15 and 30 weeks of exposure. Additionally, a 24 h time point was included to evaluate acute genotoxicity. No significant DNA damage was observed after 24 h or 15 weeks of exposure. However, a notable increase in DNA tail intensity was detected following 30 weeks, as shown in Figure 2A. This observation suggests that genotoxicity may not be an initiating event in the carcinogenic process but rather a downstream consequence of cell transformation. Transformed cells typically exhibit inherent genomic instability, which predisposes them to further genetic alterations.

**Figure 2:**
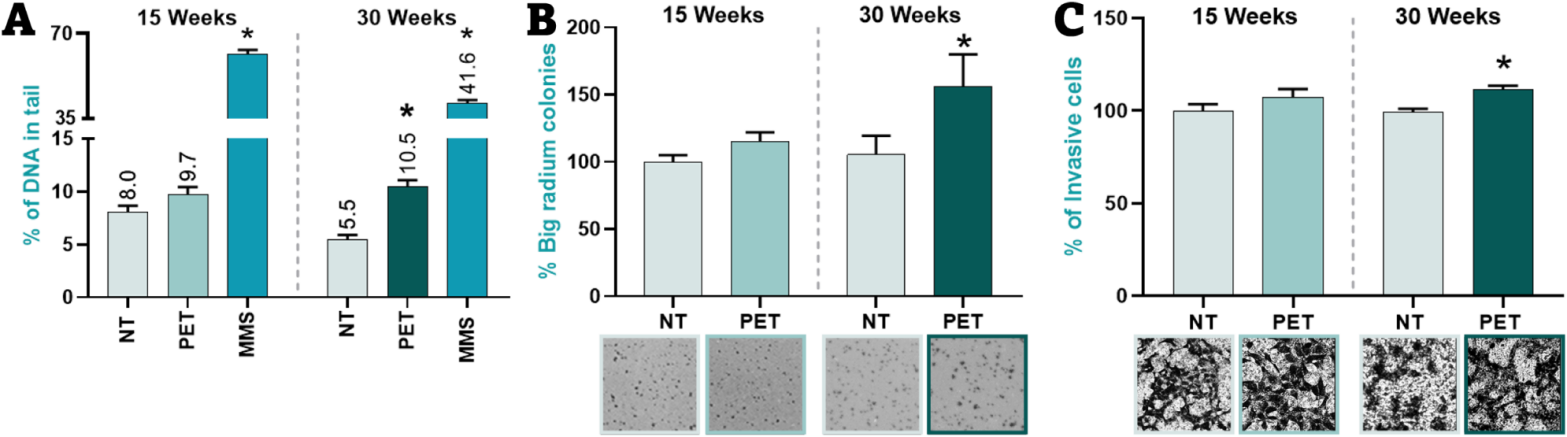
Long-term phenotypical effects of PET-NPLs in BEAS-2B cells. (A) Genotoxic damage, (B) anchorage-independent growth ability, and (C) invasive potential. Passage-paired negative control (NT) and methylmethanesulfonate (MMS, positive control).

Two additional studies from our group have explored the genotoxic potential of PET-NPLs from the same source, although using different cells and acute exposure regimes. No evidence of genotoxic effects was detected in Villacorta et al.^21^ when THP-1 cells were treated with PET-NPLs for 3 h at a range of concentrations from 5 to 50 μg/mL. In contrast, Alaraby et al.^40^ reported in vivo genotoxic effects, showing that PET-NPLs induced DNA damage in D. melanogaster hemocytes when larvae were exposed to 100 and 500 μg/g of food for 24 h.

In the literature, we can find other studies reporting the genotoxicity of PET-NPLs derived from other sources. For example, Roursgaard and collaborators^16^ found a concentration-dependent increase in DNA strand breaks in Caco-2 and HepG2 cells, measured by the alkaline comet assay, with statistically significant results obtained from 63 ng/mL after 3 h of PET-NPLs exposure. In another study involving degraded PET-NPLs, DNA damage in HepG2 cells was assessed by using the comet assay. After a 72 h treatment with PET-NPLs at concentrations ranging from 10 to 400 μg/mL, the cells exhibited significant dose-dependent genotoxic damage.^48^ Additionally, da Silva Brito and collaborators^61^ showed genotoxicity in HaCaT cells after a 6 h treatment at 100 μg/mL, as determined in this case by using the micronuclei assay. Lastly, Alzaben et al.^43^ reported that PET-NPLs induced genotoxic effects in A549 cells following a 3 h exposure at the highest concentration tested (125 μg/mL). Overall, the data indicate that PET-NPLs exhibit genotoxic properties, although with variability attributable to different doses and times of exposure used. This highlights the importance of applying long-term treatments to more accurately assess how this genotoxic potential affects health after prolonged exposure.

### PET-NPLs Chronic Exposure Increases Cells’ Anchorage-Independent Growth

During transformation, cells undergoing epithelial-to-mesenchymal transition (EMT) acquire mesenchymal traits, enabling anchorage-independent growth. In epithelial tissues, this shift promotes cell attachment to extracellular matrix (ECM) components like collagen rather than the basement membrane, driving ECM remodeling and facilitating tumor dissemination.^24,62^ In this context, the soft agar assay effectively models this cellular process and serves as a robust indicator of malignant transformation in vitro. In this study, the soft agar assay revealed no significant effects after 15 weeks of PET-NPLs exposure. However, after 30 weeks of exposure, although the total number of colonies remained unchanged, there was a significant increase in the size of the colonies, as illustrated in Figure 2B. The number of colonies exceeding 25 μm in size significantly increased compared to nontreated cells. This enlargement in colony size indicates enhanced cellular growth and survival under nonadherent conditions, suggesting the acquisition of malignancy-associated characteristics.^63^ Studies have consistently demonstrated that substances promoting larger colonies in this assay are often associated with increased cancer progression.^64^

Although no other studies have yet evaluated the effects of long-term exposure to PET-NPLs on anchorage-independent growth, our group previously tested PS-NPLs alone and in combination with arsenic in MEF cells, also obtaining positive results.^65,66^ In the first study, prolonged exposure to PS-NPLs over 24 weeks resulted in a significant increase in the level of colony formation. In the second study, while 12 weeks of PS-NPL exposure alone did not induce positive results in the soft agar assay, cotreatment with arsenic did.

### Cells Become More Invasive after Long-Term PET-NPLs Exposure

A critical step in metastasis and cancer progression is the acquisition of invasive potential. Tumor cells gain the ability to breach tissue barriers by degrading basement membranes and the ECM, which facilitates their infiltration into secondary tissues.^24,67^ Consequently, the capacity for invasion is a key biomarker for advanced and aggressive cancer phenotypes, reflecting the progression toward malignancy and metastatic potential. In this study, 15 weeks of continuous exposure of BEAS-2B cells to PET-NPLs did not reveal significant changes in invasiveness compared with passage-matched controls. However, after 30 weeks of exposure, a notable increase in invasive potential was observed, as depicted in Figure 2C. This increase in invasiveness correlates with the findings of the other carcinogenicity biomarkers tested. Therefore, BEAS-2B cells are not phenotypically transformed after 15 weeks of PET-NPLs exposure, but they reach a carcinogenic phenotype after 30 weeks.

Like studies on anchorage-independent growth, there is a lack of research on the invasiveness of cells exposed to prolonged PET-NPLs treatments. However, it is important to highlight our previous results on MEFs treated with PS-NPLs for extended periods, both alone and in combination with arsenic. Our findings revealed that, analogous to the results observed in anchorage-independent growth, extended exposure to PS-NPLs for 24 weeks led to a significant increase in cellular invasiveness. In contrast, 12 weeks of PS-NPL exposure increased the invasive potential of the cells under coexposure settings.^24^ Overall, the data suggest that prolonged exposure is essential for the manifestation of carcinogenic effects associated with NPLs and that these long-term outcomes should be further studied.

### Transcriptomic Effects of PET-NPLs Long-Term Exposure

After the phenotypical characterization of the long-term exposed BEAS-2B cells, we then moved on to evaluate the impact of PET-NPLs at the transcriptomic level. Transcriptomic analysis, encompassing gene expression profiling, pathway enrichment, and biomarker discovery, is instrumental in the early detection of cancer.^68^ Gene expression profiling can be conducted on cells and tissues—including lung—to measure cancer-related gene activity and/or mechanisms of action.^69^ These methodologies can also be effectively applied to in vitro cultures, providing valuable molecular insights into cellular transformation. Therefore, RNA-seq was performed to monitor transcriptomic alterations associated with the observed phenotypic response at three distinct time points: the acute stage (24 h of exposure), the nonphenotypically transformed stage (15 weeks), and the transformed stage (30 weeks of exposure).

### Transcriptomic Alterations Confirm the Transformation of the Cells after 30 Weeks of PET-NPLs Exposure

An in-depth assessment of the transcriptomic state of the cells transformed after 30 weeks of exposure was carried out by evaluating the number of DEGs, the enriched terms related to cancer, and a panel of genes of interest. A total of 6085 DEGs were found (3176 downregulated and 2909 upregulated, Figure 3A). After GSEA on the Disease Ontology (DO) collection was performed, 33% of the enriched terms were related to cancer (Figure 3B). Regarding gene expression levels, we examined a set of genes obtained by merging those associated with the most common natural mutations in lung carcinoma^70^ with the top 10 genes comentioned for lung cancer according to the Enrichr software. As shown in Figure 3C, the data displayed significant deregulation of 9 genes out of the set. Importantly, we found significant upregulation of three well-known oncogenes: *KRAS*, *MET*, and *RET*. RAS family variants are the most frequent hotspots for mutations in human cancers and are usually associated with a poor prognosis. Furthermore, *KRAS* mutations, which keep the gene in its active state, are commonly found in lung cancer.^71^ *MET* is known as mesenchymal-epithelial transition factor, thereby enabling tumor transformation and maintenance when activated.^72^ Finally, *RET* is overexpressed in nonsmall cell lung cancer, frequently in younger nonsmoking patients.^73^

**Figure 3:**
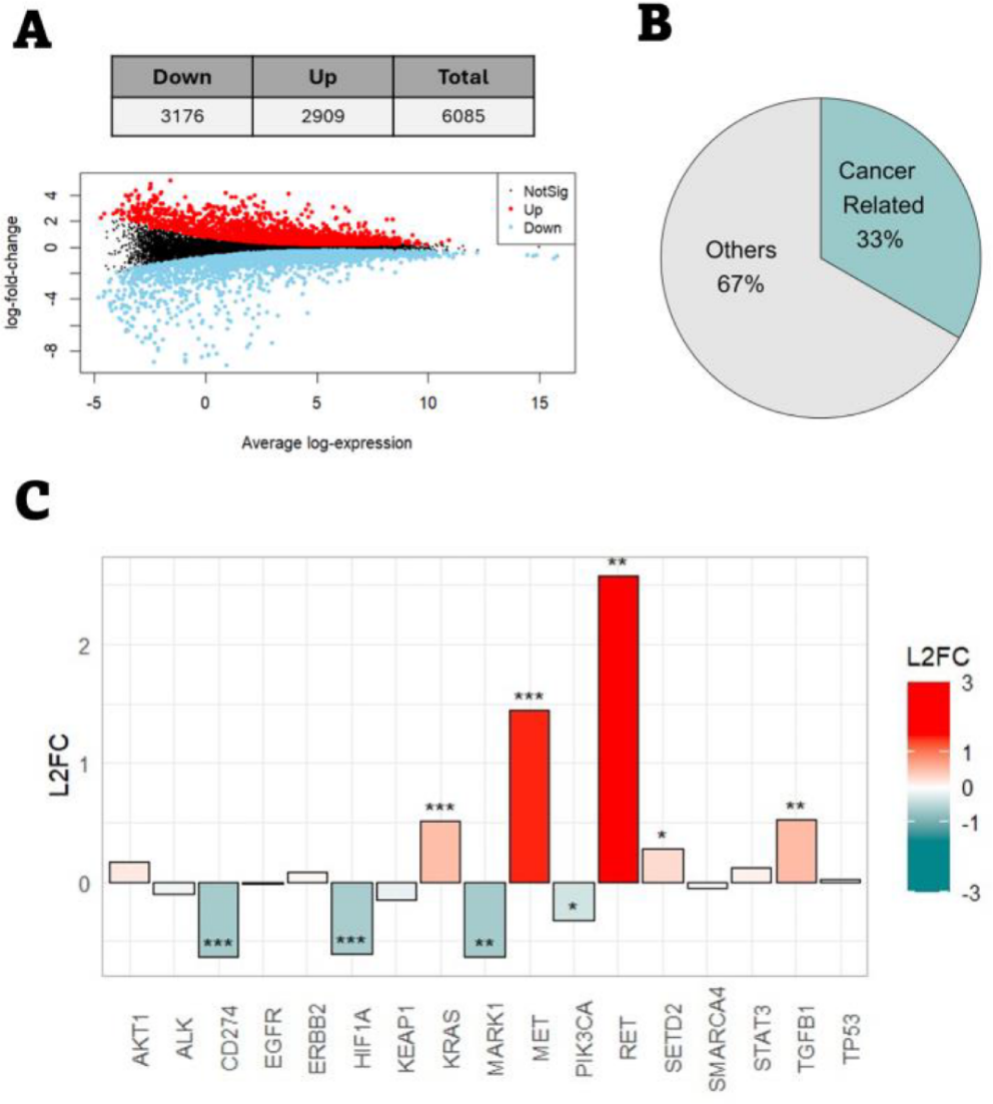
BEAS-2B transcriptomic state after 30 weeks of PET-NPLs exposure. (A) Number of DEGs (downregulated, upregulated, and total) and mean-difference plot showing the distribution of DEGs’ expression. (B) Percentage of enriched terms from DO collection corresponding to cancer-related or other terms. (C) Fold change for the genes related to the most common lung cancer mutations and the top 10 genes co-mentioned with lung cancer expressed as log2-fold-change (L2FC) values.

Additionally, we identified the 18 most frequently mutated genes published by The Cancer Genome Atlas Research Network in a comutation list containing whole-exome sequencing of 230 lung adenocarcinomas.^68^ From those 18 genes, 7 were significantly dysregulated in our data, including *KRAS, MET, NF1, PIK3CA, RB1, RIT1,* and *U2AF1*. Seven more genes were unmapped, and 4 were not differentially expressed (data available upon request).

### Transcriptomic Analysis at Different Time Points Shows the Carcinogenic Progression

Upon confirmation of cellular transformation at week 30, both at phenotypical and transcriptomic levels, earlier stages of exposure (24 h and 15 weeks) were analyzed to gain deeper insights into the progression of the transformation process. First, the number of DEGs was studied at each time point (30 weeks, 15 weeks, and 24 h). An increment in the number of DEGs over the time of exposure was observed, including an increment in both fold-change values and significance (Figure 4A). 295 DEGs were shared between the 15 and 30 weeks of exposure (Figure 4B) representing a high proportion of DEGs from the 15-week time point. Second, a deeper exploration of the enriched pathways obtained at 30 weeks of exposure by ORA and GSEA was performed. In addition, lung carcinogenic pathways alongside GO pathways sharing genes were selected and compared with enriched terms after 15 weeks of exposure (Figure 4C).

**Figure 4:**
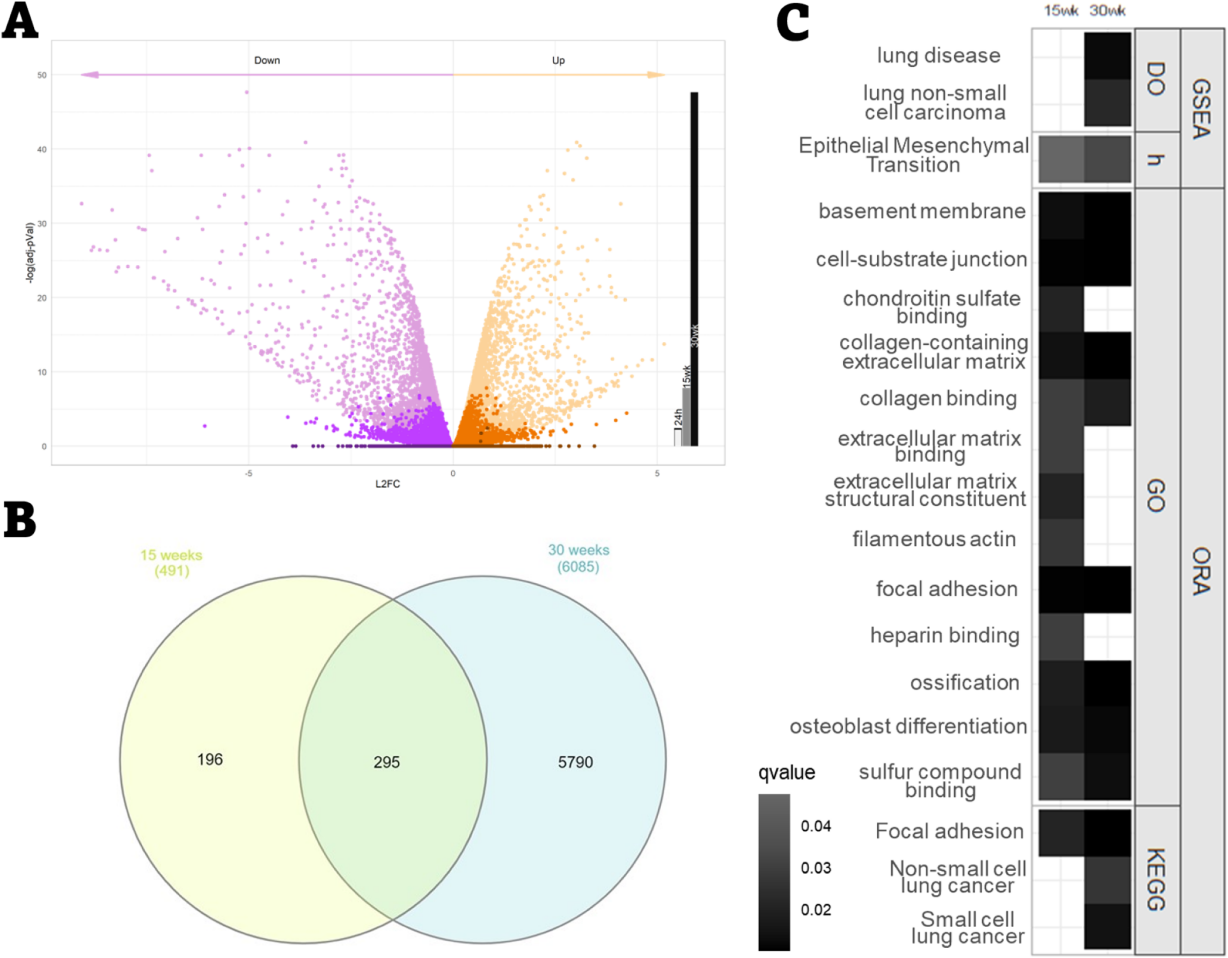
Transcriptomic progression during chronic exposure to PET-NPLs. (A) Number of DEGs after 24 h,15 weeks, and 30 weeks of exposure from darker to lighter color, respectively. Significance is not represented. (B) Venn diagram illustrating shared DEGs between 15 (yellow) and 30 weeks (blue) of exposure (C) Lung-carcinogenesis-related enriched terms found after 15 and 30 weeks of exposure. Intensity is correlated with the significance level.

The gradual increase observed in the amount of DEGs over time suggested a general progression of the transformation process. When inspecting carcinogenic-related ORA-enriched terms after 30 weeks of exposure, terms related to lung carcinogenesis were observed (“*nonsmall cell lung cancer*” and “*small cell lung cancer*”). However, none of these terms were overrepresented in the earlier stage of the analysis (15 weeks of exposure). Conversely, terms related to the cell surface, cell membrane, focal adhesion, or compound binding were present at both time points, with higher enrichment at 15 weeks. When inspecting GSEA results, the terms “*lung disease*”, “*lung nonsmall cell carcinoma*”, and “e*pithelial to mesenchymal transition*” (EMT) were found to be significant after 30 weeks, with EMT being the only pathway enriched at 15 weeks.

Both analysis results (ORA and GSEA) were concordant with the hypothesis of gradual transformation progress. After 15 weeks of exposure, terms associated with cell–substrate junctions (“*focal adhesion*”, “*basement membrane*”, “*cell–substrate junction*”, “*collagen-containing extracellular matrix*”, “*collagen binding*”, and “*epithelial to mesenchymal transition*”) were dysregulated. At this time point, the cells still did not show any carcinogenic phenotypical features, according to our data. Nonetheless, when the treatment was extended to 30 weeks, terms associated with lung cancer and lung disease appeared, along with carcinogenic phenotypical features. Overall, results from transcriptomic analyses and from phenotypical endpoints were in concordance, suggesting a carcinogenic state after 30 weeks of exposure not observable after 15 weeks, although a progression could be inferred from the transcriptomic data (see Figure S2 for a schematic summary of the findings).

Acute transcriptomic data reported in the bibliography with BEAS-2B cells exposed to PS-NPLs include the enrichment of pathways related to oxidation–reduction processes, regulation of the MAPK cascade, kinase activity, cell surface, extracellular space, anchoring junctions, adherens junctions, focal adhesion, integrin binding, small cell lung cancer, or proteoglycans in cancer, among others,^74^ concordant with the pathway enrichment results collected in this manuscript. Few data have been collected for transcriptomic data reported under long-term NPLs exposure conditions. However, in concordance with our data, gene panels targeting EMT genes were dysregulated after PS-NPLs exposure.^66^

### Mapping of Transcriptomic Data to Lung Cancer Pathways

To further confirm the transformation progress, we compared expression levels of genes annotated in the ″nonsmall cell lung cancer” pathway (KEGG pathway with identifier hsa05223) throughout the exposure period (Figure 5). This pathway was chosen as it was significantly dysregulated after 30 weeks of exposure and because of its biological relevance. In this figure, genes are represented as boxes, and each one is divided into two parts: the left side represents fold-change in gene expression levels after 15 weeks of exposure compared to passage-matched controls, whereas the right side of the box represents equivalent data after 30 weeks of exposure. The results show a relevant dysregulation of the pathway at different points, including the upregulation of the previously mentioned oncogenes: KRAS, MET, and RET (also called KIF5B-RET). No differences in gene expression levels were found for EGFR and EML4ALK oncogenes after 30 weeks of exposure, although a slight, nonsignificant fold increase was observed at week 15. Further research will be needed to explore the pathways related to the dysregulated oncogenes; nonetheless, these results point toward potential mechanisms of action leading to PET-NPLs-induced lung carcinogenesis.

**Figure 5:**
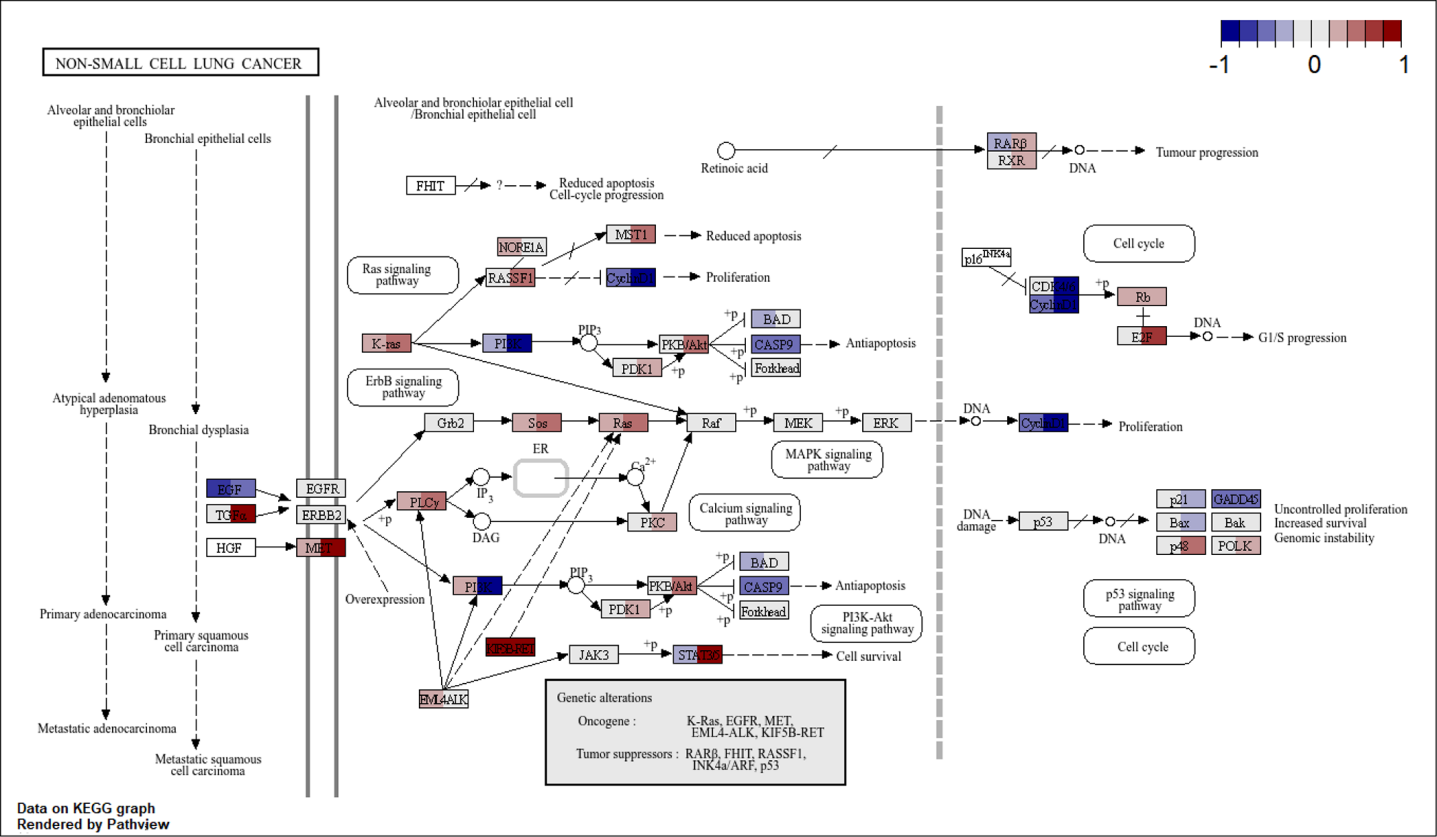
Mapping of transcriptomic data to the nonsmall cell lung cancer pathway. The figure displays fold changes in gene expression levels for annotated genes in the hsa05223 pathway from the KEGG database. Gene boxes are split in two parts: the left side corresponds to the fold-change value at 15 weeks of exposure, and the right side corresponds to the fold-change value at 30 weeks of exposure.

### Identification of Biomarkers for PET-NPLs-Induced Lung Cell Transformation

With the aim of providing a set of possible biomarkers, three gene panels were generated: (i) genes associated with the EMT pathway according to the hallmarks collection; (ii) a custom list combining the top 100 genes comentioned with the lung cancer term according to Enrichr, along with genes from a manual curation of the bibliography; and (iii) a histone panel generated by HistoneDB 2.0, as representative of genome dysregulation. Genes included in each panel were filtered by selecting the ones that followed a trend in their expression levels from 15 to 30 weeks of exposure. The 24 h time point was also included in the analysis as a point of comparison to the acute effects of PET-NPLs. As displayed in Figure 6A1, 27 genes matched EMT filtering conditions (upregulated: *TGFBR3, CDH2, IGBP2, FGF2, ITGA2, NT5E, PMP22, DKK1, ENO2, FBLN1,* and *TNC*; downregulated: *BASP1, LRP1, OXTR, LGALS1, EFEMP2, IGFBP3, GPC1, GJA1, MYLK, SAT1, SERPINH1, ADAM12, FBN1, COL1A1,* and *VCAN*), while 16 genes matched the filtering conditions of the lung cancer list (upregulated: *RET, ITGB8, IGF1R, MET, SP1, RB1, BMPR2, KRAS, CD44, FN1,* and *KRT19*; downregulated: *CCNA2, JUN, MUC1, CCND1,* and *SNAI2*) (Figure 6A2). From the histones analyzed, the vast majority were underexpressed (*H2AX, H2AC17, H2AC19, H2AC20, H2BC13, H2AC15, H3C1, H1-1, H4C1,* and *H4C6*, Figure 6B), suggesting that genome transcription is enhanced, especially after 30 weeks of exposure.

**Figure 6:**
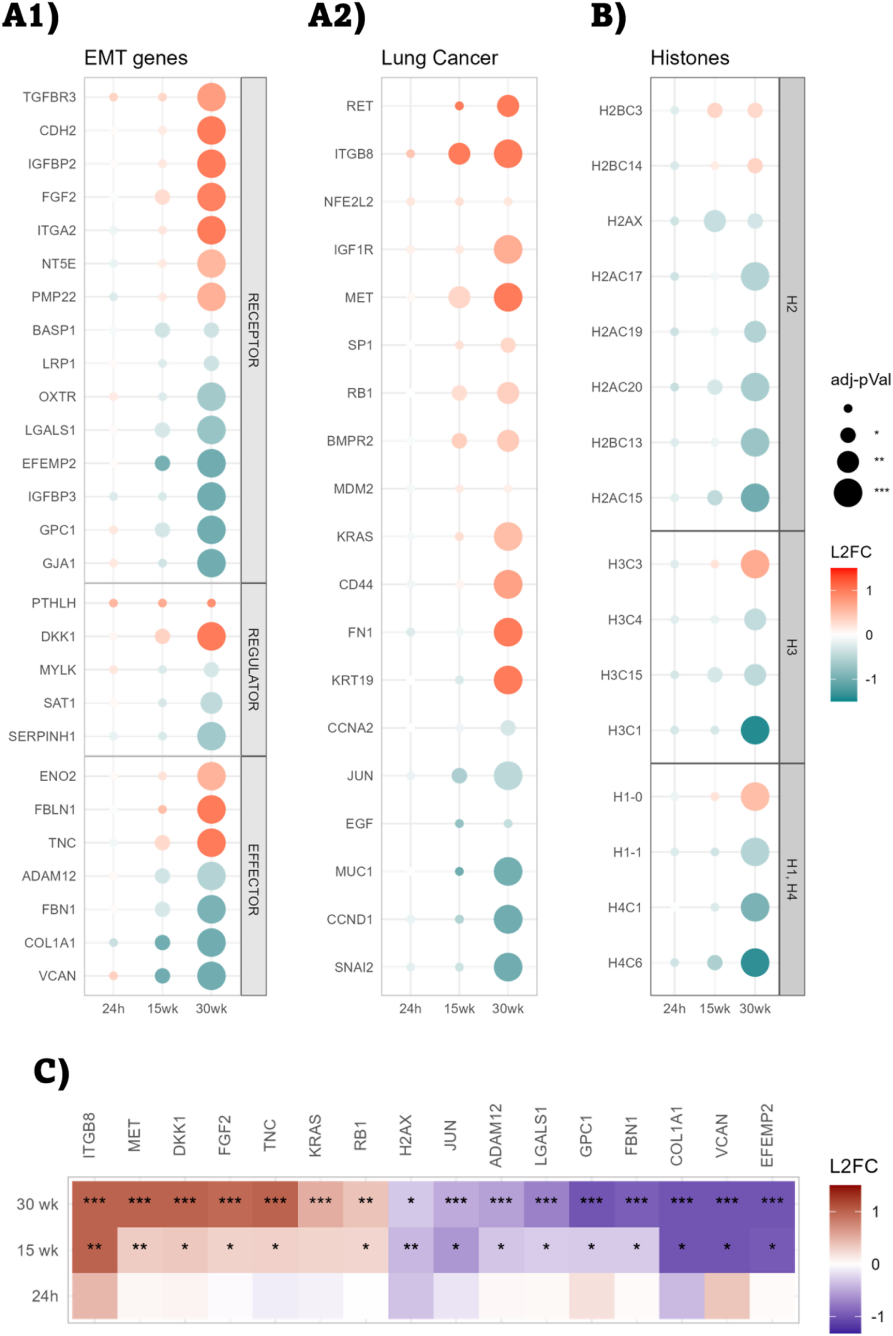
Gene expression values for genes following a trend in their expression levels during chronic exposure. Gene expression values are represented as log fold-change values (L2FC). (A1) Genes mapping EMT hallmark collection classified by their cascade position (receptor, regulator, or effector). (A2) Genes mapping the top 100 genes comentioned with lung cancer term according to Enrichr, along with manually annotated genes. (B) Histone panel according to Histone DB 2.0. (C) Putative biomarker genes that might act as early detectors of the transformation phenotype.

Combining the information from the three gene panels, we could identify 16 genes significantly dysregulated at the two long-term time points (Figure 6C). Therefore, these 16 genes were selected as putative biomarkers of lung cell transformation. From this set of biomarkers, 10 genes came from the EMT list (*FGF2, LGALS1, EFEMP2, GPC1, DKK1, TNC, ADAM12, FBN1, COL1A1,* and *VCAN*) and 4 genes from the lung cancer list (*ITGB8, MET, RB1,* and *JUN*). Additionally, *KRAS* was added to the set of biomarkers due to its biological relevance in lung cancer,^71^ and *H2AX* was selected due to its significance and capability to predict lung tumorigenic processes.^75^

Overall, we have identified a set of informative biomarkers with the potential to facilitate the characterization of the in vitro tumoral phenotype, supporting and even substituting the evaluation of certain carcinogenic hallmarks. Additionally, the biomarkers follow a trend of expression during the exposure and are significantly dysregulated early in the carcinogenic process—before phenotypical transformation—suggesting that they might act as early detectors of transformation. However, further research is required to provide a conclusion on their predictive value.

In summary, this work provides the first description of the long-term effects of real-life PET-NPLs on the respiratory system. Inhalation is one of the main routes for MNPLs exposure; therefore, the lungs are a primary target and could be seriously affected by continuous exposure, leading to bioaccumulation. It is essential to explore the detrimental effects, including carcinogenicity, of continuous MNPLs exposure to fully understand the health risks posed by their widespread presence in the environment. We have addressed this critical gap by developing a novel system to mimic chronic exposure of lung target cells to subtoxic doses of true-to-life PET-NPLs. This system relies on a multiendpoint analysis to inform on the carcinogenic potential of the exposure and explore the mechanisms involved. With this approach, information on different outcomes is organized and combined, potentially setting the basis for developing an in vitro-based ITS for NPLs with regulatory value.

Through this study, we observed that PET-NPLs induce a tumorigenic phenotype in long-term exposed BEAS-2B cells, as evidenced by their increased genome instability, genotoxic damage, anchorage-independent growth, and invasive potential—all of which are supported by significant alterations in the transcriptome, recapitulating the genomic dysregulation observed in cancer cells. Besides the in-depth characterization of the tumoral phenotype, we monitored phenotypical and transcriptomic changes at different times of exposure. Thanks to this approach, we could identify potential mechanisms of action and biomarkers that should be explored in future research, as this knowledge can help establish the pathway of causal linkages between a molecular initiating event and the final carcinogenic adverse outcome linked to chronic NPLs exposure.

These findings show the relevance of further exploring long-term effects with approaches that mimic chronic exposure scenarios. Importantly, carcinogenicity should be included as an outcome to be studied in the battery of assays for MNPLs risk assessment. The data in the literature and from our research show that there are reasonable grounds for concern regarding the carcinogenic effects of long-term MNPLs exposure. While there are still limitations to be addressed (dose–responses, variable polymer types, sizes, or shapes), the collection of this data should be a priority, with the aim of providing better protection to the general population and the environment from the potential health impacts of MNPLs.

## Supplementary Material



## Data Availability

The data discussed in this publication have been deposited in NCBI’s Gene Expression Omnibus under accession number GSE291738 and are publicly available as of publication. Any additional information required to reanalyze the data reported in this paper is available from the lead contact upon request.

## Notes

PET-NPLs characterization and a diagram illustrating the carcinogenic potential of PET-NPLs in BEAS-2B cells (PDF)

## References

[1] Plastics Europe. Plastics – the fast Facts 2023. accessed October 30, 2024. https://plasticseurope.org/knowledge-hub/plastics-the-fast-facts-2023/.

[2] Geyer R., Jambeck J. R., Law K. L. (2017). Production,
use, and fate of all
plastics ever made. Sci. Adv..

[3] Wright S. L., Kelly F. J. (2017). Plastic and human
health: A Micro Issue?. Environ. Sci. Technol..

[4] Amato-Lourenço L. F., dos Santos Galvão L., de Weger L. A., Hiemstra P. S., Vijver M. G., Mauad T. (2020). An emerging class of air pollutants:
Potential effects of microplastics to respiratory human health?. Sci. Total Environ..

[5] García-Rodríguez A., Gutiérrez J., Villacorta A., Arribas Arranz J., Romero-Andrada I., Lacoma A., Marcos R., Hernández A., Rubio L. (2024). Polylactic acid nanoplastics (PLA-NPLs) induce adverse effects on
an *in vitro* model of the human lung epithelium: The
Calu-3 air-liquid interface (ALI) barrier. J.
Hazard. Mater..

[6] Le V. G., Nguyen M. K., Nguyen H. L., Lin C., Hadi M., Hung N. T. Q., Hoang H. G., Nguyen K. N., Tran H. T., Hou D., Zhang T., Bolan N. S. (2023). A comprehensive review of micro-
and nano-plastics in the atmosphere: Occurrence, fate, toxicity, and
strategies for risk reduction. Sci. Total Environ..

[7] Luo D., Chu X., Wu Y., Wang Z., Liao Z., Ji X., Ju J., Yang B., Chen Z., Dahlgren R., Zhang M., Shang X. (2024). Micro- and nano-plastics in the atmosphere: A review of occurrence,
properties and human health risks. J. Hazard.
Mater..

[8] Dris R., Gasperi J., Mirande C., Mandin C., Guerrouache M., Langlois V., Tassin B. (2017). A first overview
of textile fibers,
including microplastics, in indoor and outdoor environments. Environ. Pollut..

[9] Wang Y., Huang J., Zhu F., Zhou S. (2021). Airborne microplastics:
A review on the occurrence, migration and risks to humans. Bull. Environ. Contam. Toxicol..

[10] Vattanasit U., Kongpran J., Ikeda A. (2023). Airborne microplastics:
A narrative
review of potential effects on the human respiratory system. Sci. Total Environ..

[11] Jenner L. C., Rotchell J. M., Bennett R. T., Cowen M., Tentzeris V., Sadofsky L. R. (2022). Detection of microplastics in human lung tissue using
μFTIR spectroscopy. Sci. Total Environ..

[12] Jiang Y., Han J., Na J., Fang J., Qi C., Lu J., Liu X., Zhou C., Feng J., Zhu W., Liu L., Jiang H., Hua Z., Pan G., Yan L., Sun W., Yang Z. (2022). Exposure to microplastics in the upper respiratory
tract of indoor and outdoor workers. Chemosphere.

[13] Issue No. 45: Plastic Tobacco Filters. A problematic and unnecessary plastic impacting the environment and human health | UNEP - UN Environment Programme. https://www.unep.org/resources/perspective-series/issue-no-45-plastic-tobacco-filters-problematic-and-unnecessary. accessed October 30, 2024.

[14] Domenech J., Villacorta A., Ferrer J. F., Llorens-Chiralt R., Marcos R., Hernández A., Catalán J. (2024). In vitro cell-transforming
potential of secondary polyethylene terephthalate and polylactic acid
nanoplastics. J. Hazard. Mater..

[15] Domenech J., Annangi B., Marcos R., Hernández A., Catalán J. (2023). Insights into the potential carcinogenicity
of micro-
and nano-plastics. Mutat. Res., Rev. Mutat.
Res..

[16] Roursgaard M., Hezareh Rothmann M., Schulte J., Karadimou I., Marinelli E., Møller P. (2022). Genotoxicity of particles from grinded
plastic items in Caco-2 and HepG2 cells. Front.
Public Health.

[17] Mastrangelo G., Fedeli U., Fadda E., Milan G., Lange J. (2002). H. Epidemiologic
evidence of cancer risk in textile industry workers: a review and
update. Toxicol. Ind. Health.

[18] Chen Q., Gao J., Yu H., Su H., Yang Y., Cao Y., Zhang Q., Ren Y., Hollert H., Shi H., Chen C., Liu H. (2022). An emerging
role of microplastics
in the etiology of lung ground glass nodules. Environ. Sci. Eur..

[19] Tollefsen K. E., Scholz S., Cronin M. T., Edwards S. W., de Knecht J., Crofton K., Garcia-Reyero N., Hartung T., Worth A., Patlewicz G. (2014). Applying adverse
outcome pathways (AOPs) to support
integrated approaches to testing and assessment (IATA). Reg. Toxicol. Pharmacol..

[20] Halappanavar S., Mallach G. (2021). Adverse outcome pathways
and *in vitro* toxicology strategies for microplastics
hazard testing. Curr. Opin. Toxicol..

[21] Villacorta A., Rubio L., Alaraby M., López-Mesas M., Fuentes-Cebrian V., Moriones O. H., Marcos R., Hernández A. (2022). A new source
of representative secondary PET nanoplastics. Obtention, characterization,
and hazard evaluation. J. Hazard. Mater..

[22] Nanogenotox. Nanogenotox: The project | Anses - Agence nationale de sécurité sanitaire de l’alimentation, de l’environnement et du travail. https://www.anses.fr/en/system/files/nanogenotox_deliverable_5.pdf. (accessed on 15 January 2022).

[23] Villacorta A., Cazorla-Ares C., Fuentes-Cebrian V., Valido I. H., Vela L., Carrillo-Navarrete F., Morataya-Reyes M., Mejia-Carmona K., Pastor S., Velázquez A., Arribas Arranz J., Marcos R., López-Mesas M., Hernández A. (2024). Fluorescent
labeling of micro/nanoplastics for biological applications with a
focus on “true-to-life” tracking. J. Hazard. Mater..

[24] Barguilla I., Maguer-Satta V., Guyot B., Pastor S., Marcos R., Hernández A. (2023). In vitro approaches to determine the potential carcinogenic
risk of environmental pollutants. Int. J. Mol.
Sci..

[25] Collins A., Møller P., Gajski G., Vodenková S., Abdulwahed A., Anderson D., Bankoglu E. E., Bonassi S., Boutet-Robinet E., Brunborg G. (2023). Measuring DNA modifications
with the comet assay: A compendium of protocols. Nat. Protoc..

[26] R Core Team R: A Language and environment for statistical computing. R Foundation for Statistical Computing, 2023. https://www.R-project.org/. (accessed 2025-April-02).

[27] Posit Team. RStudio: Integrated development environment for R; Posit Software; PBC, Boston, MA. 2023. https://posit.co/products/enterprise/team/. accessed October 30, 2024.

[28] Chen S., Zhou Y., Chen Y., Gu J. (2018). Fastp: An ultra-fast
all-in-one FASTQ preprocessor. Bioinformatics.

[29] Liao Y., Smyth G. K., Shi W. (2019). The R package Rsubread is easier,
faster, cheaper and better for alignment and quantification of RNA
sequencing reads. Nucleic Acids Res..

[30] Chen Y., Lun A. T. L., Smyth G. K. (2016). From reads to genes to pathways:
differential expression analysis of RNA-Seq experiments using Rsubread
and the edgeR quasi-likelihood pipeline. F1000Research.

[31] Leek J. T., Johnson W. E., Parker H. S., Jaffe A. E., Storey J. D. (2012). The sva
package for removing batch effects and other unwanted variation in
high-throughput experiments. Bioinformatics.

[32] Ritchie M. E., Phipson B., Wu D., Hu Y., Law C. W., Shi W., Smyth G. K. (2015). limma powers differential
expression analyses for RNA-sequencing
and microarray studies. Nucleic Acids Res..

[33] Law C. W., Chen Y., Shi W., Smyth G. K. (2014). Voom: Precision
weights unlock linear model analysis tools for RNA-seq read counts. Genome Biol..

[34] Yu G., Wang L. G., Han Y., He Q. Y. (2012). ClusterProfiler:
An R package for comparing biological themes among gene clusters. OMICS: J. Integr. Biol..

[35] Wickham, H. ggplot2 Elegant Graphics for Data Analysis; Springer-Verlag: New York, 2016.

[36] Histone Database 2.0. accessed November 28, 2024. https://histdb.intbio.org/.

[37] Kuleshov M. V., Jones M. R., Rouillard A. D., Fernandez N. F., Duan Q., Wang Z., Koplev S., Jenkins S. L., Jagodnik K. M., Lachmann A., McDermott M. G., Monteiro C. D., Gundersen G. W., Ma’ayan A. (2016). Enrichr: a
comprehensive gene set enrichment analysis web server 2016 update. Nucleic Acids Res..

[38] Annangi B., Villacorta A., Vela L., Tavakolpournegari A., Marcos R., Hernández A. (2023). Effects of
true-to-life PET nanoplastics
using primary human nasal epithelial cells. Environ. Toxicol. Pharmacol..

[39] Tavakolpournegar A., Villacorta A., Morataya-Reyes M., Arranz J. A., Banaei G., Pastor S., Velázquez A., Marcos R., Hernández A., Annangi B. (2024). Harmful effects of true-to-life nanoplastics derived
from PET water bottles in human alveolar macrophages. Environ. Pollut..

[40] Alaraby M., Villacorta A., Abass D., Hernández A., Marcos R. (2023). The hazardous impact
of true-to-life PET nanoplastics
in Drosophila. Sci. Total Environ..

[41] Ji Y., Chen L., Wang Y., Yu Y., Wang M., Wang X., Liu W., Yan B., Xiao L., Song X. (2024). Realistic nanoplastics
induced pulmonary damage via
the crosstalk of ferritinophagy and mitochondrial dysfunction. ACS Nano.

[42] Zhang H., Zhang S., Duan Z., Wang L. (2022). Pulmonary toxicology
assessment of polyethylene terephthalate nanoplastic particles in
vitro. Environ. Int..

[43] Alzaben M., Burve R., Loeschner K., Møller P., Roursgaard M. (2023). Nanoplastics from ground polyethylene
terephthalate
food containers: Genotoxicity in human lung epithelial A549 cells. Mutat. Res., Genet. Toxicol. Environ. Mutagen..

[44] Arribas
Arranz J., Villacorta A., Rubio L., García-Rodríguez A., Sánchez G., Llorca M., Farre M., Ferrer J. F., Marcos R., Hernández A. (2024). Kinetics and toxicity of nanoplastics
in ex vivo exposed human whole blood as a model to understand their
impact on human health. Sci. Total Environ..

[45] Magrì D., Sánchez-Moreno P., Caputo G., Gatto F., Veronesi M., Bardi G., Catelani T., Guarnieri D., Athanassiou A., Pompa P. P., Fragouli D. (2018). Laser ablation as a
versatile tool to mimic polyethylene terephthalate nanoplastic pollutants:
Characterization and toxicology Aassessment. ACS Nano.

[46] Magrì D., Veronesi M., Sánchez-Moreno P., Tolardo V., Bandiera T., Pompa P. P., Athanassiou A., Fragouli D. (2021). PET nanoplastics interactions with water contaminants
and their impact on human cells. Environ. Pollut..

[47] Johnson L. M., Mecham J. B., Krovi S. A., Moreno Caffaro M. M., Aravamudhan S., Kovach A. L., Fennell T. R., Mortensen N. P. (2021). Fabrication
of polyethylene terephthalate (PET) nanoparticles with fluorescent
tracers for studies in mammalian cells. Nanoscale
Adv..

[48] Manoochehri Z., Etebari M., Pannetier P., Ebrahimpour K. (2024). *In
vitro* toxicity of polyethylene terephthalate nanoplastics
(PET-NPs) in human hepatocarcinoma (HepG2) cell line. Toxicol. Environ. Health Sci..

[49] OECD Guidelines for the Testing of Chemicals | OECD iLibrary. https://www.oecd-ilibrary.org/environment/oecd-guidelines-for-the-testing-of-chemicals_72d77764-en. accessed October 30,2024.

[50] OECD. Development Co-operation Report 2016: The Sustainable Development Goals as Business Opportunities; OECD Publishing: Paris, 2016.

[51] Annys E., Billington R., Clayton R., Bremm K. D., Graziano M., McKelvie J., Ragan I., Schwarz M., van der
Laan J. W., Wood C., Öberg M., Wester P., Woodward K. N. (2014). Advancing the 3Rs in regulatory toxicology
- Carcinogenicity testing: Scope for harmonisation and advancing the
3Rs in regulated sectors of the European Union. Regul. Toxicol. Pharmacol..

[52] Schmeisser S., Miccoli A., von Bergen M., Berggren E., Braeuning A., Busch W., Desaintes C., Gourmelon A., Grafström R., Harrill J., Hartung T., Herzler M., Kass G. E. N., Kleinstreuer N., Leist M., Luijten M., Marx-Stoelting P., Poetz O., van Ravenzwaay B., Roggeband R., Rogiers V., Roth A., Sanders P., Thomas R. S., Marie Vinggaard A., Vinken M., van de Water B., Luch A., Tralau T. (2023). New approach methodologies in human
regulatory toxicology – Not if, but how and when!. Environ. Int..

[53] Kirsch A., Dubois-Pot-Schneider H., Fontana C., Schohn H., Gaté L., Guichard Y. (2020). Predictive early gene signature during mouse Bhas 42
cell transformation induced by synthetic amorphous silica nanoparticles. Chem. Biol. Interact..

[54] Guichard Y., Savoy C., Gaté L. (2023). Can a 12-gene expression signature
predict the cell transforming potential of tumor promoting agents
in Bhas 42 cells?. Toxicol. Lett..

[55] Vales G., Rubio L., Marcos R. (2016). Genotoxic
and cell-transformation
effects of multi-walled carbon nanotubes (MWCNT) following in vitro
sub-chronic exposures. J. Hazard. Mater..

[56] Bach J., Peremartí J., Annangi B., Marcos R., Hernández A. (2016). Oxidative
DNA damage enhances the carcinogenic potential of *in vitro* chronic arsenic exposures. Arch. Toxicol..

[57] Rubio L., Bach J., Marcos R., Hernández A. (2017). Synergistic
role of nanoceria on the ability of tobacco smoke to induce carcinogenic
hallmarks in lung epithelial cells. Nanomedicine.

[58] Domenech J., de Britto M., Velázquez A., Pastor S., Hernández A., Marcos R., Cortés C. (2021). Long-term effects of polystyrene
nanoplastics in human intestinal Caco-2 cells. Biomolecules.

[59] Nymark P., Karlsson H. L., Halappanavar S., Vogel U. (2021). Adverse outcome pathway
development for assessment of lung carcinogenicity by nanoparticles. Front. Toxicol..

[60] Kirkland D., Reeve L., Gatehouse D., Vanparys P. (2011). A core *in vitro* genotoxicity battery
comprising the Ames test plus the *in
vitro* micronucleus test is sufficient to detect rodent carcinogens
and *in vivo* genotoxins. Mutat.
Res..

[61] da
Silva Brito W. A., Ravandeh M., Saadati F., Singer D., Dorsch A. D., Schmidt A., Cecchini A. L., Wende K., Bekeschus S. (2024). Sonicated polyethylene terephthalate nano- and micro-plastic-induced
inflammation, oxidative stress, and autophagy *in vitro*. Chemosphere.

[62] Janiszewska M., Primi M. C., Izard T. (2020). Cell adhesion
in cancer: Beyond the
migration of single cells. J. Biol. Chem..

[63] Mahalingaiah P. K. S., Singh K. P. (2014). Chronic oxidative stress increases growth and tumorigenic
potential of MCF-7 breast cancer cells. PLoS
One.

[64] Afify S. M., Seno M. (2019). Conversion of stem cells to cancer
stem cells: Undercurrent of cancer
initiation. Cancers.

[65] Barguilla I., Domenech J., Rubio L., Marcos R., Hernández A. (2022). Nanoplastics
and arsenic co-exposures exacerbate oncogenic biomarkers under an *in vitro* long-term exposure scenario. Int. J. Mol. Sci..

[66] Barguilla I., Domenech J., Ballesteros S., Rubio L., Marcos R., Hernández A. (2022). Long-term
exposure to nanoplastics alters molecular
and functional traits related to the carcinogenic process. J. Hazard. Mater..

[67] Friedl P., Wolf K. (2003). Tumour-cell invasion and migration: diversity and escape mechanisms. Nat. Rev. Cancer.

[68] Cancer Genome Atlas Research Network. Comprehensive molecular profiling of lung adenocarcinoma. Nature 2014, 511(7511), 543–550. 10.1038/nature13385. 25079552 PMC4231481

[69] Hijazo-Pechero S., Alay A., Marín R., Vilariño N., Muñoz-Pinedo C., Villanueva A., Santamaría D., Nadal E., Solé X. (2021). Gene expression
profiling as a potential
tool for precision oncology in non-small cell lung cancer. Cancers.

[70] Chevallier M., Borgeaud M., Addeo A., Friedlaender A. (2021). Oncogenic
driver mutations in non-small cell lung cancer: Past, present and
future. World J. Clin. Oncol..

[71] Singhal A., Li B. T., O’Reilly E. M. (2024). Targeting
KRAS in cancer. Nat. Med..

[72] Liang H., Wang M. (2020). MET oncogene in non-small
cell lung cancer: Mechanism of MET dysregulation
and agents targeting the HGF/c-Met axis. OncoTargets
Ther..

[73] Drusbosky L. M., Rodriguez E., Dawar R., Ikpeazu C. V. (2021). Therapeutic strategies
in RET gene rearranged non-small cell lung cancer. J. Hematol. Oncol..

[74] Zhang T., Yang S., Ge Y., Wan X., Zhu Y., Li J., Yin L., Pu Y., Liang G. (2022). Polystyrene nanoplastics
induce lung injury via activating oxidative stress: Molecular insights
from bioinformatics analysis. Nanomaterials.

[75] Matthaios D., Hountis P., Karakitsos P., Bouros D., Kakolyris S. (2013). H2AX a promising
biomarker for lung cancer: a review. Cancer
Invest..

