# Supplementary material for: Long-Term Exposure to Real-Life Polyethylene Terephthalate Nanoplastics Induces Carcinogenesis In Vitro
Source: Environ Sci Technol. 2025 Jun 2;59(22):10891–904. doi: 10.1021/acs.est.5c01628 (PMC12164274; doi:10.1021/acs.est.5c01628)
Supplement: Supplementary file 1 [file es5c01628_si_001.pdf]

Long-Term Exposure to Real-Life Polyethylene Terephthalate Nanoplastics Induces Carcinogenesis *in vitro*

Javier Gutiérrez-García, Raquel Egea, Irene Barguilla, Penny Nymark, Alba García-Rodríguez, Boris Guyot, Veronique Maguer-Satta, Ricard Marcos, Laura Rubio*, Alba Hernández*

J Gutiérrez-García, R. Egea, A. García-Rodríguez, R. Marcos, L. Rubio*, A. Hernández*

Group of Mutagenesis, Department of Genetics and Microbiology, Faculty of Biosciences, Universitat Autònoma de Barcelona, 08193 Cerdanyola del Vallès, Barcelona, Spain.

*Email: laura.rubio@uab,cat;

*Email: alba.hernandez@uab.cat

I. Barguilla, B. Guyot, V. Maguer-Satta,

CNRS UMR5286, Centre de Recherche en Cancérologie de Lyon, 69008 Lyon, France.

I. Barguilla, B. Guyot, V. Maguer-Satta,

Inserm U1052, Centre de Recherche en Cancérologie de Lyon, Lyon, France.

P. Nymark

Institute of Environmental Medicine, Karolinska Institutet, 17177 Stockholm, Sweden.

Summary: 3 pages, 2 figures

SUPPORTING INFORMATION

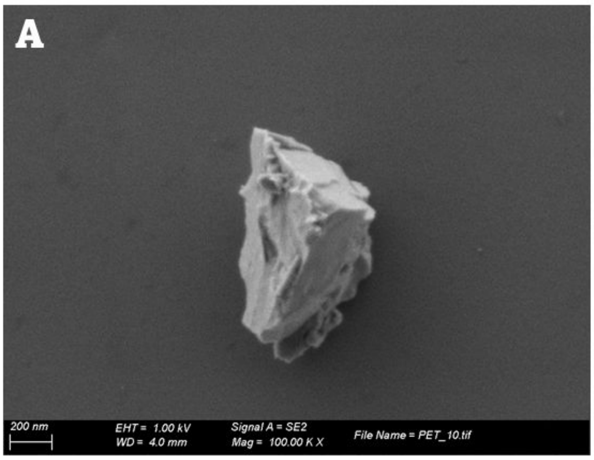

B

	Milli-Q				DMEM		
	SEM	Z-average	PDI	Z-potential	Z-average	PDI	Z-potential
PET-NPLs	176.24 ± 6.52	502.73 ± 17.112	0.667 ± 0.057	28.6 ± 0.26	420.77 ± 29.520	0.730 ± 0.067	- 15.16 ± 0.85

Figure S1. PET-NPLs characterization. (A) SEM images obtained from PET-NPLs (B) Characterization table of nanoparticles. PET-NPLs were analyzed in two different media: Milli-Q water and DMEM culture media. SEM images were analyzed using ImageJ software with Fiji extension, while DLS was used to determine hydrodynamic size and Z-potential. SEM and Z-average values are provided in nanometers (nm) while Z-potential measure is provided in millivolts (mV). PDI = Polydispersity index.

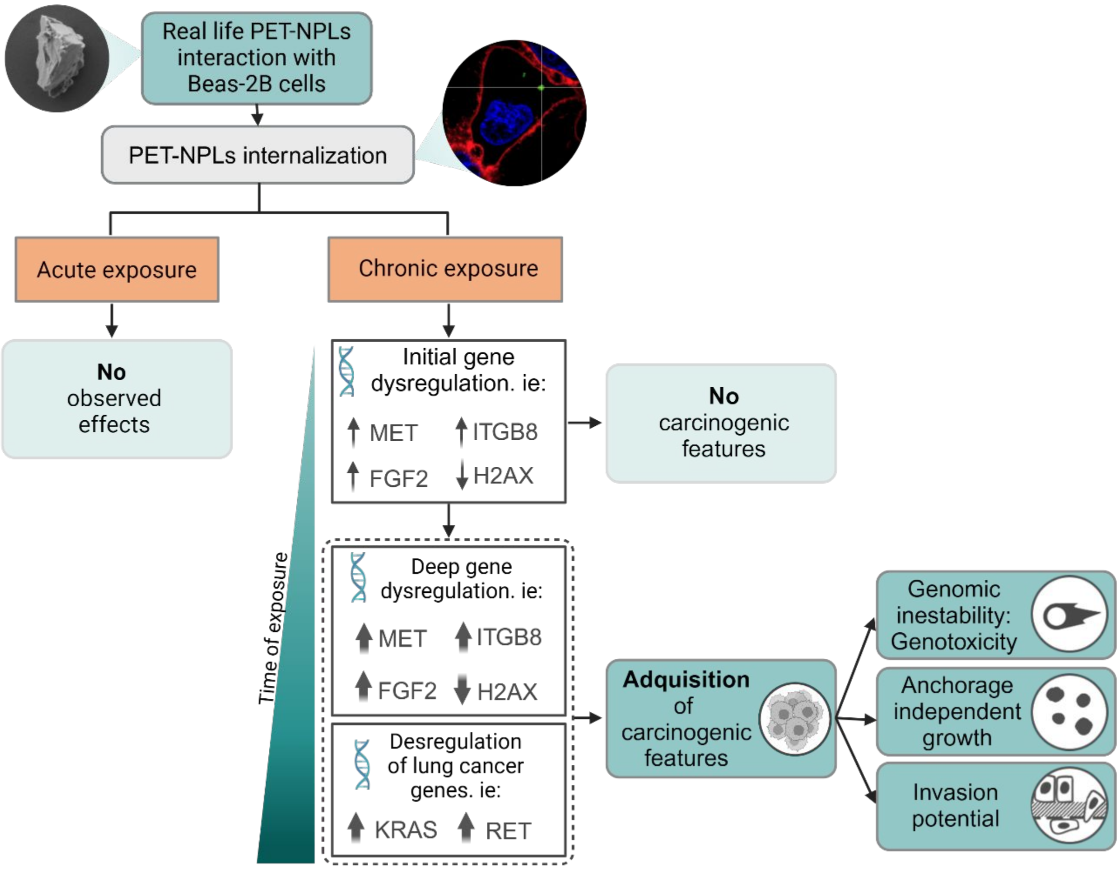

Figure S2. Diagram illustrating the carcinogenic potential of PET-NPLs in BEAS-2B cells. While acute exposure shows no observable effects, prolonged exposure induces molecular alterations that intensify over time, ultimately leading to the acquisition of a cancer-like phenotype. This is evidenced by the emergence of genomic instability, anchorage-independent growth, and increased invasive capacity.
